# Evaluating the Impact of a Pilot Nationwide Webinar Series on National Med-Peds Residents' Association Membership

**DOI:** 10.7759/cureus.18923

**Published:** 2021-10-20

**Authors:** Maximilian J Cruz, Sasha R Kapil, Allen R Friedland, Ronald J Magliola

**Affiliations:** 1 Internal Medicine, University of Cincinnati Medical Center, Cincinnati, USA; 2 Internal Medicine-Pediatrics, Indiana University, Indianapolis, USA; 3 Internal Medicine-Pediatrics, ChristianaCare, Newark, USA; 4 Internal Medicine-Pediatrics, MetroHealth Medical Center, Cleveland, USA; 5 Internal Medicine-Pediatrics, Case Western Reserve University, Cleveland, USA

**Keywords:** internal medicine-pediatrics, residency programs, nmpra, virtual recruitment, webinar, medicine, pediatrics, med-peds, medical students

## Abstract

Background: As the COVID-19 pandemic significantly reduced the ability of medical students to travel and interact directly with combined Internal Medicine-Pediatrics (Med-Peds) residency programs, medical students desiring appropriate guidance and information about Med-Peds residency training needed a national forum for information during an unprecedented virtual recruitment year.

Objective: To develop a nationally coordinated webinar series for medical students and student advisors to learn about the Med-Peds specialty for residency training to keep applicant numbers and applicant interest from significantly falling.

Methods: A national webinar series focusing on general Med-Peds information, career interests, and tailored advising was created amongst the three national Med-Peds organizations over a three-month period in Spring 2020.

Results: There was a 221% increase in medical student membership to the National Med-Peds Residents' Association (NMPRA) compared to the same months in 2017, 2018, and 2019 and no significant reduction in the Electronic Residency Application Service^®^ applications to Med-Peds programs over that same time period.

Conclusions: A national forum for medical students inquiring about the combined Med-Peds specialty can be effective in recruiting members to NMPRA and keeping interest high in Med-Peds.

## Introduction

Combined Internal Medicine-Pediatrics (Med-Peds) residency attracts students interested in the care of people and families across the age continuum, generally in primary care, hospital care, specialty care, or a combination of multiple areas [1]. Medical students often have an interest in the care of both children and adults. Even though Med-Peds is the largest combined specialty, there are only 77 programs currently across the United States, and only an estimated 8000 Med-Peds graduates since its inception in 1967. Due to this, medical students interested in Med-Peds often face a paucity of mentors and influences local to their medical school. The COVID-19 pandemic triggered cessation of medical student clinical activities at a critical time for medical students deciding on specialty preference(s) for The National Resident Matching Program® (NRMP®).

The process of recruiting medical students to the field of Med-Peds is largely dependent on mentors and advisors who have a good understanding of the nuances of combined specialties. One-third to one-half of Med-Peds applicants come from schools without affiliated Med-Peds residency programs. For this reason, an estimated 200 students per year use fourth-year electives to confirm their choice of applying to Med-Peds, as these rotations provide invaluable insight into a Med-Peds program structure and give students access to the Med-Peds mentorship and advising often not available at their home institutions. Due to the COVID-19 pandemic, many clinical rotations were significantly truncated, or entirely suspended, following guidance from the Association of American Medical Colleges (AAMC) [2]. Away rotations, including Med-Peds electives, became largely unavailable to students outside their respective host institutions.

Anticipating a significant impact on Med-Peds recruitment and a fall in applicant numbers, the Med-Peds community consisting of the National Med-Peds Residents’ Association (NMPRA), the Recruitment Committee of the Med-Peds Program Directors’ Association (MPPDA), and the American Academy of Pediatrics Section on Med-Peds (AAP-SOMP), collaborated in the Spring of 2020 to organize a virtual recruitment approach creating a national webinar series for both medical students and advisors. Our group was guided by the following aims: (I) Improve student interest in Med-Peds and maintain a stable number of applicants into our specialty; (ii) Highlight the depth and breadth of Med-Peds training and career opportunities; (iii) Standardize information regarding Med-Peds recruitment; (iv) Assuage medical student concerns about applying to Med-Peds; (v) Ensure equity between all Med-Peds programs and medical students to participate; (vi) Reduce the necessity for similar local seminars hosted by multiple, individual residency programs.

At the time of this initiative, there were no previously coordinated national, virtual recruitment series targeting medical students and advisors within the literature. This article describes a novel interactive program to foster interest in the discipline of Med-Peds associated with dramatic and unprecedented pandemic-related recruitment challenges.

## Materials and methods

Session topics and focus

**Figure 1:**
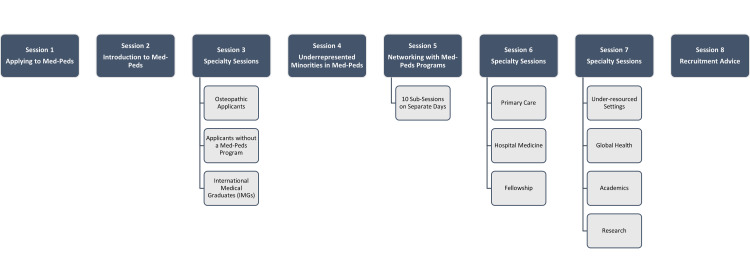
Webinar Series Topics

After discussion with all partner organizations, It was decided to tailor this series to meet the needs of fourth-year medical students considering applying to Med-Peds or to another specialty. Session topics (Figure 1) were vetted based on our aims and fell into three topic areas.

Med-Peds Information (101 Beginner and 201 Advanced Sessions)

Multiple sessions provided an overview of the field of Med-Peds at both an introductory level (for first through third year medical students with limited knowledge of Med-Peds) as well as advanced level sessions (for fourth year medical students who received deeper insights into career paths, rotation requirements, and other areas of interest to a student already familiar with Med-Peds). There was a series of “spotlight” sessions to showcase each individual Med-Peds program. During these sessions, each Med-Peds residency program was given five minutes to give a brief overview of their program and values. Of the 77 total Med-Peds programs, 75 programs participated in the spotlight sessions. At the end of each session, students were able to ask questions directly to program directors and leadership on the webinar.

Career Interests

Several sessions highlighted career pathways of Med-Peds graduates, largely designed to correct the misconception that Med-Peds graduates most often “choose” to solely practice either Medicine or Pediatrics. Visiting Med-Peds electives provide students with insight into the variability and flexibility of Med-Peds careers and allow them to witness how Med-Peds attendings integrate and work in both disciplines. Given the lack of away electives this year, our sessions provided exposure to the myriad career paths that Med-Peds offers, as well as data behind the career trends in Med-Peds. In total, over 100 unique faculty members from almost every program were involved in the webinar series.

Tailored Advising

Specialty sessions were created specifically for osteopathic applicants, international medical graduate applicants, and applicants from schools without affiliated Med-Peds programs. These applicant populations often face limited access to Med-Peds advisors, particularly advisors familiar with and capable of navigating their unique application scenarios. A session was also held specifically for medical school advisors, to update them on the field of Med-Peds, as well as answer any questions to help them better advise their respective student groups.

Logistics

Zoom and WebEx™ were the primary virtual platforms used. Panelists with expertise in the session topic were invited to present, and a facilitator was assigned to moderate each session. Given the speed with which the webinar series was constructed, the process for setting up and disseminating meeting information changed throughout, but the ultimate finalized process was as follows:

1. Zoom or WebEx™ meeting set up with appropriate security features (password protected, participants muted, participant screenshare disabled).

2. Meeting ID and password distributed to medical students registered as members of NMPRA via a member electronic mailing list.

3. Promotion of upcoming webinar(s) on social media (Twitter, Instagram) without associated login links/information. Students were encouraged to reach out directly if they did not receive the login information via email.

4. NMPRA social media and email accounts monitored daily for any students reaching out for meeting information and provided instruction on how to be notified about future webinars.

5. Meetings led by a single facilitator, with one or more panelists.

6. Meetings recorded and uploaded to the NMPRA website for asynchronous viewing.

Data

We collected data on medical students registering for NMPRA membership. We used registrations within the date range of May 22, 2020-September 2, 2020, which represent the day the webinar series was first announced and one week after the final webinar, respectively. For comparison, we pulled data for that same date range, as well as annual registrations, for each year back to 2017 (the inception of our database). We also reviewed published Electronic Residency Application Service® (ERAS®) preliminary data, as of January 13, 2021, regarding residency applicants for the 2021 application cycle [3]. After the conclusion of the webinar series, we piloted a survey to explore how applicants chose to watch the webinars. We asked them to indicate how many webinars they viewed as recorded sessions and compared this to the number they viewed as live sessions.

## Results

NMPRA membership

There was a significant increase in medical student registration for NMPRA membership in 2020 over the specified time, as well as the full calendar year versus that in the three prior years. New student registration increased by 221% over the selected range in 2020 compared to 2019. For the full year, there was a 92% increase in total annual student registration compared to 2019 (Table 1).

**Table 1:** National Med-Peds Residents' Association (NMPRA) New Member Registration

Year	New Registrations (May 22–September 2 of Respective Year)	New Registrations (Full Year)
2017	73	164
2018	89	332
2019	86	306
2020	276	587

ERAS® applications

There was a small decrease in overall applicants (nine) to the field of Med-Peds in 2021 compared to 2020, but this appears to fit typical variations seen over the four prior years (Table 2).

**Table 2:** Med-Peds Electronic Residency Application Service® Applicant Numbers (each January)

Year	Total Applicant Number
2017	865
2018	959
2019	934
2020	966
2021	957

Survey results of webinar participants

**Figure 2:**
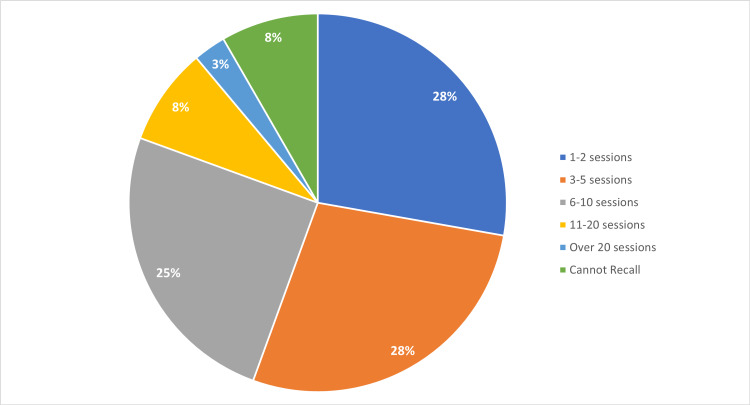
Number of Live Webinar Sessions Attended by Medical Students (n=36)

Of the 73 survey respondents, 36 (50%) chose to respond to a question regarding their live webinar attendance. Of these respondents, 56% indicated that they attended up to five sessions in a live format. One student was able to attend all sessions live (Table 3, Figure 2).

**Table 3:** Number of Live Webinar Sessions Attended by Medical Students (n=36)

Number of Sessions	Number of Medical Students
1‒2 Sessions	10
3‒5 Sessions	10
6‒10 Sessions	9
11‒20 Sessions	3
Over 20	1
Cannot Recall	3

**Figure 3:**
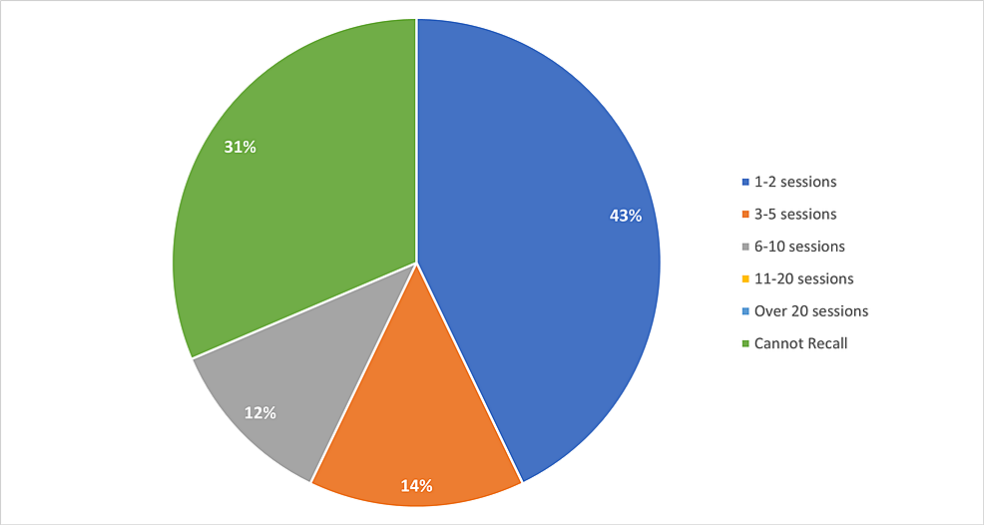
Number of Recorded Webinars Watched by Medical Students (n=35)

To understand if there was any benefit to recording the session for asynchronous viewing, medical students were also asked how many recorded webinar sessions they watched. Table 4 breaks down the number of sessions that medical students each attended. Of the 35 survey respondents who answered the question regarding their recorded webinar viewing, 67% utilized the option to review a recorded session later (Table 4, Figure 3).

**Table 4:** Number of Recorded Webinars Watched by Medical Students (n=35)

Number of Sessions	Number of Medical Students
1‒2 Sessions	15
3‒5 Sessions	5
6‒10 Sessions	4
11‒20 Sessions	0
Over 20	0
Cannot Recall	11

## Discussion

As a combined specialty, Med-Peds faces unique recruitment challenges. The COVID-19 pandemic highlighted and amplified those challenges with the suspension of clinical clerkships as well as visiting clerkships at the time when many students were finalizing which specialty(ies) to apply. The leadership of the Med-Peds field was concerned about a reduction of applicants to Med-Peds due to a limited ability to interact with students. In response, we proactively produced a national (both live and recorded) webinar series to serve as a source of accurate and reliable information regarding the specialty of Med-Peds for medical students considering applying into Med-Peds and advisors of medical students over the 2021 recruitment season. For the first time, most Med-Peds programs (75/77) used the same platform to describe their program and its culture, and over 100 faculty were involved in some way with the webinar series.

We used new membership to NMPRA as a proxy for the potential impact the webinar series had on medical student engagement and interest in Med-Peds. This webinar series drove an unprecedented significant increase in medical student enrollment in the National Med-Peds Residents’ Association (NMPRA) proximate to the webinar series (221%) and for the entire academic year (92%). Although not tracked specifically, there was an increased number of students requesting assistance with finding mentors, becoming active in the newsletter, and attending national educational Med-Peds meetings.

Both live and/or recorded sessions seemed to be utilized by half of the respondents, but live participants were better able to quantify the number of live sessions attended, and 78% (23/33) that recalled the number of live sessions attended at least three webinars and 38% (9/24) that recalled the number of recorded sessions participated in at least three webinars.

Early in the webinar series, we noted medical students created an applicant social media chat, facilitated in part by the relationships and contacts they made through our webinar series. The students also hosted their own virtual Med-Peds happy hour Zoom event prior to the interview season to network and create meaningful connections that may not have occurred otherwise.

The data from the ERAS supported stability in medical students that applied to Med-Peds [3] which exceeded our pre-webinar expectation and was within year-to-year fluctuation in medical student applications. The data from the NRMP showed for the first time that Med-Peds had a 100% fill rate in the Main Match [4].

Limitations

Despite the many successes of Med-Peds as a specialty this past recruitment season, we encountered several challenges. Early on, we relied on each meeting’s selected facilitator to set up and schedule the meeting on the virtual platform (Zoom or WebEx™) available to them. This made it difficult, at times, to retrieve and upload recordings, as we had to rely on those individuals to remember to record the session and provide links to the recording, which was often delayed (justifiably, given increased clinical and administrative demands amidst the pandemic). We also had a lesson early in the webinar series on the importance of securing the webinar rooms and not providing login information outside of a secure email list, and we quickly created stricter protocols after one session was hacked. This change in security significantly hampered our ability to fully advertise the sessions without introducing an additional step to provide individuals with specific login information and to make sure it only went to the intended participant(s).

Since the series was started urgently, the need to develop and track participants to survey was not the top priority. We attempted to survey our audience throughout the series to gain insight into the effectiveness of the series in providing students with information on Med-Peds and impacting their decisions whether to apply into Med-Peds, but that was erratic; so we then tried to survey participants at the end of the webinar series and had a low response rate. Since we were not able to collect a lot of data, we were not able to determine the demographic characteristics of medical student attendees, nor the number of unique participants session-over-session. Also, we are unable to definitively determine the impact of the webinars for students applying and ranking Med-Peds programs (i.e., identify medical students who became more committed to Med-Peds as a direct result of the webinars), so we can only presume that our webinars helped by maintaining comparable applicant numbers compared to prior years.

## Conclusions

Our nationally coordinated webinar series allowed Med-Peds organizations to effectively collaborate to provide medical students with valuable information and exposure regarding the field of Med-Peds at a time when an unprecedented pandemic severely limited more traditional experiences and exposures that students rely on to solidify their decision to apply into Med-Peds. The success of this series on such short notice shows the utility of using new platforms to engage with medical students, and we anticipate building on our experiences to create a similar recruitment platform next year. While causality cannot be inferred, Med-Peds had, for the first time, a 100% fill rate in the Main Match. With more time to plan for the 2022 recruitment season, we anticipate a more robust evaluation of the impact and outcomes of our future webinar series.
